# Introducing Ten to Men, the Australian longitudinal study on male health

**DOI:** 10.1186/s12889-016-3697-2

**Published:** 2016-10-31

**Authors:** Jane Pirkis, John Macdonald, Dallas R. English

**Affiliations:** 1 Centre for Mental Health, Melbourne School of Population and Global Health, The University of Melbourne, Melbourne, 3010 Australia; 2 Centre for Epidemiology and Biostatistics, Melbourne School of Population and Global Health, The University of Melbourne, Melbourne, 3010 Australia; 3 School of Science and Health, Western Sydney University, Penrith, 2751 Australia

## Abstract

We are very pleased to introduce Ten to Men, the Australian Longitudinal Study on Male Health. Ten to Men is, to our knowledge, the largest national all-male cohort study in the world. It involves 15,988 males who were aged between 10 and 55 years when we recruited them in 2013/14. Together, the articles in this collection provide an overview of the study’s methods, examples of some of the key questions it can answer, and guidance for researchers wishing to use it. Perhaps most importantly, the articles demonstrate the enormous potential Ten to Men has to make a real difference to our understanding of male health and the factors that influence it.

## Background

Male health is a significant issue which has only recently begun to receive the attention that it warrants. The health profile of males in Australia mirrors that in other Western countries. Australian males have shorter life expectancies than their female counterparts for a variety of reasons (e.g., young males are significantly more likely to die from accidental causes) [1]. Males are more likely than females to experience some of the health problems that account for the greatest disease burden in Australian society (e.g., cardiovascular disease) [2]. Males also have higher rates of behaviours that place them at risk for particular health problems (e.g., alcohol consumption) and are less likely to visit health professionals [1]. Research on the social determinants of male health and the way these influence health outcomes, behaviours and health service use has been fairly limited.

In response to this, the Australian Government released the National Male Health Policy [3] in 2010, as part of its commitment to improving the health of Australian males and making the health system more responsive to their needs. The policy identified the need for research that could help to build a strong evidence base on male health that could be used to inform policies, programs and initiatives. It called for a large-scale longitudinal study as the cornerstone of this evidence base.

In 2011, the Department of Health funded us to establish Ten to Men, recruit the cohort and conduct the baseline wave of data collection (Wave1). Ten to Men was designed as a longitudinal study to ensure that it could contribute to knowledge about the factors that influence male health, with a particular emphasis on social determinants of health (e.g., where boys and men live, study, work and/or take part in recreational activities). It ambitiously targets boys as well as men so that the influence of key transitions (e.g., reaching puberty, leaving school, beginning work, becoming a father for the first time, retiring) can be examined. It complements other, more established longitudinal studies in Australia (e.g., the Australian Longitudinal Study on Women’s Health [ALSWH] [4], the Longitudinal Study of Australian Children [LSAC] [5]), but its exclusive focus on males allows us to ‘drill down’ into factors that may be of particular relevance to male health. The large sample size allows us to consider particular sub-groups of males who may be at heightened risk of poor health outcomes (e.g., those living in regional areas).

## The articles in this collection

We hope that the articles in this collection will whet readers’ appetites for Ten to Men. The first article, by Currier et al. [6], sets the scene by providing an overview of the study. It describes the process that led us to our 15,988 males. We knocked on the doors of all of the households in areas selected via a stratified random sampling technique, and invited all of the eligible males within each household to participate. We deliberately over-sampled in areas of Australia outside major cities (referred to as inner and outer regional areas) to ensure that we had sufficient numbers of participants in these areas to undertake meaningful analyses. We collected data on a range of constructs (physical health, mental health and wellbeing, health behaviours, social determinants of health, and health service utilisation and health knowledge), using self-complete questionnaires for participants aged 15 to 55 and the parents of boys aged 10 to 14. We also conducted interviews with the boys themselves. We achieved a 35 % response fraction and high questionnaire/interview completion rates.

The article by Spittal et al. [7] provides more detail about the sampling strategy used in Ten to Men, exploring the implications of four of its key elements (stratification, multi-stage sampling, clustering and sample weights) for the analysis of Wave 1 data. This article notes that estimates of prevalence will be biased if the hierarchical nature of the data and sample weights are ignored, whereas estimates of association will be less likely to be affected. They use the examples of weight and smoking status in the cohort to illustrate these points.

The next seven articles draw on the Wave 1 data to answer a range of important research questions. Kavanagh et al. [8] profile men with and without disabilities on a range of socio-demographic and health-related variables and show that the former are much more likely to experience social and economic disadvantage. For example, they are more likely to live in tenuous circumstances and find it difficult to make ends meet. They are also less likely to have a job, and, if they are employed, are less likely to be performing skilled roles and more likely to be working fewer hours than they would prefer to be. They have lower levels of social support and community participation, and worse physical and mental health. As Kavanagh et al. [8] point out, these results underscore the need to ensure that health and social policy supports men with disabilities.

LaMontagne et al. [9] take a different angle on employment and working conditions, focusing particularly on psychosocial job quality. They observe that men who have low levels of control over their jobs, find their work too demanding or complex, have minimal job security, are faced with unfair payment practices, work long hours and/or do shift work have significantly poorer mental health and wellbeing than men who do not work under these conditions. The more of these adverse conditions men confront in their working lives, the worse they fare. This has significant implications for workplace reform; improvements in psychosocial job quality would undoubtedly benefit working men themselves, and there is evidence that employers would also benefit from having an emotionally healthier workforce.

Currier et al. [10] consider a broader range of life stressors and examine the extent to which these are related to suicidal thinking in men. More specifically, they consider whether life stressors exert an influence on suicidal thinking independently of mental disorders. They find that certain stressful life events (e.g., serious family conflict, difficulty finding a job, legal troubles, major loss of property, break-up of a relationship and serious personal injury) significantly increase the risk of suicidal thinking in the absence of mental disorders, but when these events take place in the context of such disorders (particularly depression) the risk is amplified. This suggests that suicide prevention strategies, whether they are delivered in clinical or population settings, should not only focus on mental health problems but should also address what is happening in men’s lives.

Senaratna et al.’s [11] article shifts the focus to sleep apnoea. They demonstrate that the prevalence of this condition increases as men age. They also show that it is associated with indicators of poorer self-rated physical and mental health and wellbeing, and it clusters with a number of other chronic conditions. It is also associated with unemployment. Senaratna et al. [11] suggest that preventive efforts might capitalise on the fact that sleep apnoea occurs co-morbidly with a number of other conditions, and that interventions designed to modify lifestyle-related factors (e.g., smoking, alcohol consumption, low levels of physical activity and overweight) might not only reduce sleep apnoea in men but other chronic conditions as well.

Koelemeyer et al. [12] consider one such chronic condition, namely diabetes. They show that diabetes is relatively common in adult males, and that those who have been diagnosed with diabetes are more likely to be socio-economically disadvantaged than their peers who have not. The former group also fare worse when it comes to other physical and mental health conditions, and are more likely to rate their own health as poor. Koelmeyer et al. [12] suggest that their findings may have implications for how to improve the targeting of diabetes screening in men.

Schlichthorst et al. [13] explore an issue that is often hidden, namely sexual difficulties in men. They note that this issue is more common than many people might expect; over 50 % of men experience at least one sexual difficulty that lasts at least 3 months in a given year. Sexual difficulties present in young men as well as older men, and are associated with health factors (e.g., poor self-rated health, having a disability, having a mental health condition) and lifestyle factors (e.g., smoking, consuming alcohol at harmful levels, taking drugs). Schlichthorst et al. [13] reflect on their findings and conclude that discussions about sexual health and sexual functioning should constitute part of a routine health check for men of all ages.

In the final paper, Schlichthorst et al. [14] move away from looking at particular conditions, and consider instead men’s health service use. In particular, they consider the likelihood of men consulting with a general practitioner (GP) in a given year or having an annual health check-up. They report that 81 % of men consult with a GP annually, but only 39 % have an annual health check. An interesting mix of factors is predictive of both types of health service use. On the one hand, low levels of use are associated with indicators of good health (e.g., those who rate their own health as excellent are less likely to visit a GP). On the other hand, low levels of use are also associated with indicators of poorer health (e.g., those who consume alcohol at harmful levels are less likely to have an annual health check). Either way, it would seem to be the case that there are missed opportunities for proactive discussions about health promotion and disease prevention.

## The capacity of Ten to Men to increase knowledge about male health

The articles in the collection tell an important story. Some common themes emerge from the individual sets of data analysis. In particular, it is evident that, irrespective of the health issue, men who are disadvantaged financially or geographically are the most affected. In addition, and not unrelatedly, conditions cluster together both within and across physical health and mental health domains.

This set of articles provides a taster of the enormous capacity of Ten to Men to address policy-relevant questions about male health. There is so much more that can be done with the Wave 1 data. There are numerous other exposures and outcomes that can be considered (e.g., the relationship between masculinity and suicidal thinking [Pirkis J, Spittal MJ, Keogh L, Mousaferiadis T, Currier D: Masculinity and suicidal thinking, submitted 2016]). Much of our work to date has involved data from the men aged 18–55 in the cohort, but there are many questions that can be asked about the boys (aged 10–14) and the young men (aged 15–17). There are also multiple other analyses that can be done (e.g., capitalising on our sampling approach to examine intra-household relationships between boys and men). Although we are beginning to explore some of these ideas and have others in the pipeline, the list is vast and we are keen for others to interrogate the data.

The Wave 1 data have their limitations, however. Their cross-sectional nature data make it difficult to determine the causal direction of the observed relationships. So, for example, one interpretation of the findings in Kavanagh et al’s [8] article is that having a disability can lead to poor employment prospects and insecure economic circumstances in men. Another interpretation is that men who experience this sort of socio-economic disadvantage are at greater risk of disability. As subsequent waves of data become available, Ten to Men will really start to come into its own because it will be possible to determine the chronology of events which, in turn, will allow causal inferences to be drawn with greater certainty. Wave 2 data collection has just been completed, and these data will be available in early 2017. The plan is that subsequent waves will be conducted every 2–3 years. Again, our intention is to make these data as broadly available as possible, to maximise the utility of Ten to Men.

Data are available to researchers via the Australian Data Archive (https://www.ada.edu.au/ada/01311). We encourage those who are interested in accessing Ten to Men data to visit the study page at the archive, or the study website (www.tentomen.org.au) for further information on the data request process, as well as full documentation of the resource including Wave 1 and 2 questionnaires, Wave 1 databooks and the Data User Manual.

## Conclusions

We know we are biased, but we believe that Ten to Men could be a game-changer in terms of how we understand male health and its complex antecedents and consequences. Internationally, there is a growing movement which is giving male health greater prominence, and considering what needs to change to improve it. The consistent message from the articles in this collection is that social determinants play a key role in male health, suggesting that although some policy and practice reform needs to occur within the health sector, more – much more – needs to take place in sectors outside health that have an influence on the physical and mental wellbeing of boys and men. These sectors include, but are obviously not limited to, the employment, education, social services, housing and justice sectors.
